# Association between youth blood pressure and exposure to pediatric fruit and vegetable prescriptions

**DOI:** 10.1038/s41390-024-03671-w

**Published:** 2024-12-10

**Authors:** Amy Saxe-Custack, David Todem, Jenny LaChance, Jean Kerver, James Anthony

**Affiliations:** 1 Charles Stewart Mott Department of Public Health, Michigan State University–Hurley Children’s Hospital Pediatric Public Health Initiative, Flint, MI USA; 2 Department of Epidemiology and Biostatistics, College of Human Medicine, Michigan State University, East Lansing, MI USA

## Abstract

**Background:**

Health impacts of pediatric fruit and vegetable prescription programs (FVPPs) are unclear. This study assessed whether exposure to an FVPP that provided $15 produce prescriptions during pediatric visits was associated with differences in child diet, food security, physical activity, weight status, and blood pressure.

**Methods:**

This cross-sectional analysis included data from caregiver-child dyads with varying levels of exposure to the FVPP. Dyads completed surveys at pediatric offices. Trained research assistants measured height and weight of children and recorded blood pressure through chart review. Causal inference analyses using propensity score adjustments compared outcomes of exposure groups.

**Results:**

680 dyads enrolled. Youth who received ≥1 prescription (exposed) reported greater physical activity compared to youth who received no prescriptions (unexposed). Blood pressure percentiles were lower among exposed when compared to unexposed youth (63.273 versus 75.060 for SBP; 71.472 versus 77.548 for DBP); and fewer exposed children recorded elevated blood pressure when compared to unexposed (0.141 versus 0.343 for SBP; 0.199 versus 0.344 for DBP; and 0.286 versus 0.531 overall). Similar findings were obtained using duration as a measure of exposure.

**Conclusion:**

Youth exposed to the FVPP experienced greater physical activity and healthier blood pressure. Findings may indicate novel health-promoting effects of pediatric FVPPs.

**Impact:**

- When compared to youth with no exposure, youth exposed to a pediatric fruit and vegetable prescription program recorded greater physical activity and healthier blood pressure.
- Youth with high exposure (≥24 months) to the fruit and vegetable prescription program experienced greater physical activity and healthier blood pressure when compared to youth with low exposure (<24 months).
- This extends evaluation of pediatric fruit and vegetable prescription programs beyond feasibility and preliminary effectiveness to indicate potential of such programs in positively influencing physical activity and blood pressure of participating youth.
- Findings indicate novel health-promoting effects of pediatric fruit and vegetable prescription programs.

## Introduction

Regular consumption of fruits and vegetables during childhood and adolescence promotes health,^1–3^ however, most young people struggle to reach minimum daily fruit and vegetable recommendations.^4–7^ Instead, ultraprocessed foods dominate total energy intake among youth in the United States (US).^8^ Because ultraprocessed food consumption is associated with increased adiposity^9^ as well as adverse health conditions, including cardiovascular disease and type 2 diabetes,^10^ there are urgent calls for public health measures to encourage fresh foods among youth. Health care clinics are now partnering with community-based organizations to introduce fruit and vegetable prescription programs (FVPPs).^11–14^ These programs vary in design and scope. Most include health provider-issued prescriptions that local food retailers accept as payment for fresh fruits and vegetables. Previous research has supported the positive impacts of FVPPs on diet and health of adults.^13–17^ However, few have examined whether, and to what degree, exposure to pediatric FVPPs influences nutrition among youth and their families.^18–21^ Moreover, the impact of such programs on children’s weight status and blood pressure has not been well-studied.^22^

In February 2016, a large pediatric clinic in a low-income city introduced Michigan’s first pediatric FVPP. This program included pediatrician-issued prescriptions for fresh produce during all office visits. Prescriptions were redeemable for fresh produce at either a local farmers’ market or mobile market that moved throughout the city. Caregivers whose children were exposed to this year-round FVPP felt it was effective in improving child diet and household food security.^23^ After expansion of the same FVPP to a second pediatric clinic, preliminary research found an association between improvements in food security and dietary behaviors of children and program exposure.^18,19^ A third pediatric clinic began issuing prescriptions in March 2021. The purpose of the current study was to compare dietary intake, food security, physical activity, weight status, and blood pressure across varying levels of pediatric FVPP exposure at three participating clinics. The number of fruit and vegetable prescriptions (FVRx) received served as the primary measure of FVPP exposure with the exposed group including those children who received at least one prescription and the unexposed group including those who received no prescriptions. A secondary measure of exposure was the length of time children were exposed to the FVPP divided into high exposure (average 40.70 months) and low exposure (average 7.76 months).

## Methods

The sample for this cross-sectional study consisted of caregiver-child dyads seen within three partnering pediatric clinics located in Flint, Michigan. Caregiver-child dyads (one per household) were enrolled based on child exposure to an FVPP that provided one $15 fruit and vegetable prescription to patients at each office visit. Exposure was caregiver-reported and clinic-verified: (1) number of prescriptions the participating child received since program initiation, and (2) a record of FVPP program exposure stratified by greater than or equal to 24 months (high exposure) or less than 24 months (low exposure).

Dyads were recruited from pediatric clinics serving demographically similar patient populations. Clinic 1 is a university-affiliated pediatric clinic that introduced Michigan’s first pediatric FVPP in February 2016. Clinic 2 is one of the largest private-practice pediatric clinics in Flint serving over 3000 young patients. The FVPP was introduced at clinic 2 in August 2018 to assess the feasibility and preliminary effectiveness of the FVPP at a second clinic site.^19^ Clinic 3 is the largest pediatric clinic in Genesee County, with Flint as its urban center. Expansion of the FVPP to clinic 3 occurred in March 2021 and substantially increased program reach among vulnerable children and families living in Flint. Clinic 3 has a comparable patient population to clinics 1 and 2, serving only children in need who are residents of Genesee County.

**Fig. 1:**
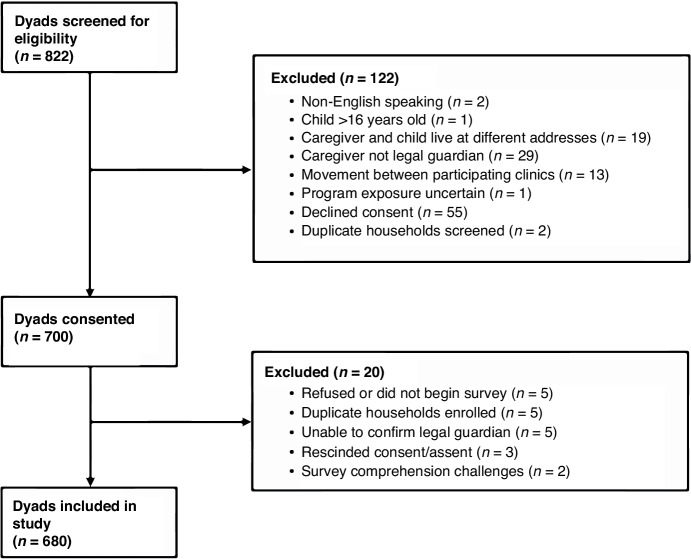
Consort diagram of the recruitment process.

To ensure sufficient data for analyses, a sample size calculation was completed. Using a one-way ANOVA analysis for three groups, with alpha of 0.05 and assuming a small-medium effect size of 0.15, 567 total dyads were required to result in a power of 0.90. Inclusion criteria for all participants were child age between 8 and 16 years and active patient status at one of three partnering clinics. Exclusion criteria for all participants included caregiver or child not English speaking (<2% of the Flint population); legal guardian not present at enrollment; child assent refused; sibling previously enrolled (one caregiver and one child per household); and movement between participating clinics (Fig. 1).

### Theoretical model

In design and approach, the current study was grounded in the theoretical framework of Bandura’s Social Cognitive Theory (SCT), which suggests that behavior is explained by a three-stage, dynamic model between personal factors, environmental factors, and behavior.^24,25^ This study’s SCT framework involved collaborations with partnering physicians and local agricultural leaders to support environmental change and social learning. Since children are generally guided by parents in their dietary intake, environmental factors, such as access to fruits and vegetables as well as caregiver modeling were of critical importance.^26,27^ Consistent with SCT, self-efficacy is central to behavior change. Accordingly, self-efficacy for consuming fruits and vegetables, which refers to one’s judgment over their capability to do so, is important to change dietary behavior.^28^ By supporting the study with the theoretical framework of SCT, it was suggested that pediatrician issuance of a prescription for fruits and vegetables to children during office visits would promote self-efficacy to consume healthy foods while positively influencing health-promoting behaviors.^29^

### Intervention description

FVPP processes were built into the electronic medical record (EMR) systems, with prescriptions recorded and printed per practitioner order and tracked monthly. FVPP vendors included the downtown Flint Farmers’ Market (FFM) and Flint Fresh, a mobile market and food hub that offered locally grown, home-delivered, fresh produce boxes (flintfresh.org). Vendors treated $15 prescriptions as vouchers redeemable only for fresh fruits and vegetables.

### Data collection procedures

From February 2021 through June 2022, following consent and assent procedures, caregiver-child dyads separately completed surveys to assess sociodemographic characteristics, dietary intake, food security, and physical activity. Sections of this survey were previously pilot-tested.^11^ All self-reported data was collected in the clinic using a secure digital platform (Research Electronic Data Capture, or REDCap) available to dyads using iPads. Trained research assistants provided detailed instructions regarding the study and survey completion. Research assistants also measured child height and weight following standardized procedures and extracted systolic blood pressure (SBP) and diastolic blood pressure (DBP) measures and date through medical chart review.

### Defining exposure groups

Considering the importance of dietary patterns in childhood on long-term dietary behaviors^30–32^ alongside evidence that suggests adolescence is the period when food insecurity has the greatest potential for negative impacts on diet,^33^ data collection and analyses focused specifically on children and adolescents ages 8–16 years. The primary measure of program exposure was the number of FVRx received by the participating child, and the secondary measure was duration in the program. Two subgroups were specified based on these measures of FVPP exposure. For the primary measure of exposure, the exposed group included children who received one or more FVRx in contrast to the unexposed group who received no FVRx. For the second measure, participants were divided into the high exposure (≥24 months) and low exposure (<24 months). The specification based on duration in the program was guided by previous literature which suggests that repeated exposure to novel foods is a key mechanism through which child food acceptance occurs. Specifically, six or more exposures have consistently been shown to produce liking and intake of vegetables.^34–39^ Fruit and vegetable tracking has indicated that most pediatric patients (8–18 years of age) reach this threshold after approximately 24 months (2 years) of exposure to the current FVPP.

### Primary outcome measures

#### Child dietary patterns

Eating behaviors were assessed via child report using the 41-item Block Kids Food Screener (BKFS), chosen for low respondent burden and acceptable psychometric values.^40^ Dietary analysis, using Block Online Analysis System, provided nutrient estimates and number of servings by food groups. This data was used to determine mean daily intake of total fruits and vegetables, total vegetables, total fruits, and whole fruits.

#### Household food security

Household food security was assessed by caregiver report using the US Household Food Security Module: Six Item Short Form (National Center for Health Statistics) via caregiver report.^41^ The sum of affirmative responses to six questions served as the household’s raw score. Food security status was assigned based on a calculated raw score (0–1 = high/marginal food security; 2–4 = low food security; 5–6 very low food security).

#### Child physical activity

Physical activity was assessed via child-report using the eight-question Patient-Reported Outcomes Measurement Information System (PROMIS) Physical Activity questionnaire for children and adolescents, which was developed and validated to assess patient-reported outcomes for clinical research and practice.^42^

#### Child weight status

Trained research assistants measured children’s height and weight without shoes or heavy outer garments. On each occasion, two measures were made and recorded, and the averages were used in analyses. Height was measured to the closest 0.1 cm using a portable stadiometer. Weight was measured to the closest 0.2 kg on a digital platform scale accurate to 200 kg. Research assistants entered data electronically into REDCap. Height and weight of the caregiver were self-reported and used to calculate body mass index (BMI) as a moderating variable for child's BMI percentile. Child BMI, which correlates with more expensive and direct measures of body fat,^43^ was calculated from child height and weight (weight (kg)/[height (m)]^2^). BMI was then categorized into percentiles by sex and age to serve as an indicator of overweight and obesity.^44^

#### Child blood pressure

Trained research assistants at each clinic extracted systolic and diastolic blood pressure values from medical charts. Approximately 92% of blood pressure measurements were taken by trained clinic staff and recorded in medical charts on the same day as study enrollment. These values were categorized relative to percentiles based on age, sex, and measured height.^45^

#### Demographics

Caregivers self-reported household income, age, education, race, height and weight, city of residence, and participation in food assistance programs at study enrollment. Assisted by caregivers and research assistants, youth self-reported age, sex, race, and city of residence.

### Data analyses

Basic descriptive statistics were generated for demographic variables. Inferential analyses, based on simple t-tests and chi-square tests, were further conducted to examine distributional differences in these characteristics across the exposure groups. Under the primary measure of exposure, all but 1 pediatric patient out of 376 at clinics 1 and 2 were perfectly classified into the exposed arm with the average number of FVRx received equal to 4.55 ± 4.29 (range 1 to 50 FVRx, first quartile = 2 FVRx, Median = 3 FVRx, and third quartile = 6 FVRx). Among the 304 patients at clinic 3, 169 were classified in the exposed arm with the average number of FVRx received equal to 1.96 ± 1.35 (range 1–9 FVRx, first quartile = 1 FVRx, Median = 2 FVRx, and third quartile = 2 FVRx). Using the second measure of exposure, all pediatric patients at clinics 1 and 2 (*n* = 376) were perfectly classified into the high exposure arm with an average length of exposure to the FVPP equal to 40.70 ± 6.14 months (range of 30.62–53.65 months, first quartile = 35.82 months, Median = 40.23 months, and third quartile = 44.94 months). Similarly, all patients at clinic 3 (*n* = 304) were perfectly classified into the low exposure arm with an average length of exposure to the FVPP equal to 7.76 ± 4.28 months (range 0–14.12 months, first quartile = 3.88 months, Median = 8.21 months, and third quartile = 11.73 months).

Because of the observational nature of the current study, causal inference analyses were conducted to compare baseline measures of the exposure groups. Specifically, a balancing strategy based on propensity scores (PS) was used to mitigate potential differences in confounders at baseline between comparison groups.^46^ These scores were estimated from an elaborated parametric logistic model that included variables that potentially confound the relationship between the exposure to FVPP and study endpoints (e.g., child’s level, caregiver’s level covariates, and functions thereof). Estimated scores from this analysis were then used to obtain an unbiased estimator of the average treatment effect (ATE) of FVPP exposure on both primary and secondary study endpoints. Specifically, the augmented inverse probability weights (AIPW) method, a combination of inverse probability weighting and regression adjustment methods were used to perform doubly robust estimation of the ATE. This method provides unbiased estimates of the ATE when either the propensity or the measurement model is mis-specified.^47^ Estimates and confidence intervals of potential outcome means (POM) for each exposure group, as well as those of the mean and risk differences between the two exposure groups, were computed. Because the empirical estimates of the standard errors of the AIPW estimates are known to be biased, bootstrap-based estimates of the standard errors and confidence intervals were generated.

Selection of potential confounders was determined based on prior knowledge in setting up the most plausible causal diagram. From this conceptual exercise, a minimal sufficient adjustment set was identified yielding observed confounders and proxies for unobserved confounders. The following covariates were selected as potential confounders for the effect of the exposure to FVPP on study endpoints: age of the child, sex of the child, race of the child, caregiver’s education, caregiver’s age, caregiver’s BMI, participation in the Supplemental Nutrition Assistance Program (SNAP), participation in free/reduced school lunch programs, city of residence, and caregiver’s overall perceptions of pediatric health care score. These variables provided a reasonable balance of the two study groups derived from the primary and secondary measures of exposure. The measurement models under the AIPW method were specified using parametric regression models (e.g., logistic and linear regression models) with potential confounders varying with the study endpoints.

Handling of missing data was based on complete case analysis due to negligible (less than 3%) missing data rates across all endpoints and covariates. That is, missing values in the outcome or the propensity score model were removed from the analysis. All analyses were performed with the use of SAS software, version 9.4 (SAS Institute).

This study was approved by Michigan State University Institutional Review Board and informed consent was obtained from all participants. The study was registered through clinicaltrials.gov [ID: NCT04767282] on February 23, 2021.

## Results

A total of 680 caregiver-child dyads (1360 participants) enrolled into the study across three clinics between February 2021 and June 2022 (Fig. 1). Characteristics of participants are described in Table 1 by primary exposure to the FVPP (number of prescriptions received). Mean age of children who received at least one prescription (exposed) was slightly higher than mean age of children unexposed to the FVPP (12.5 years of age versus 11.98 years of age, *p* = 0.0191). Additionally, caregivers of exposed children were older than caregivers of unexposed children (40.49 years of age versus 38.16 years of age, *p* = 0.0048) and reported more desirable scores related to perceptions of their child’s pediatric health care^48^ (85.81 versus 79.84, *p* < 0.0001). Other sociodemographic characteristics of caregivers and children did not vary appreciably.

**Table 1:** Baseline demographic characteristics by exposure group (Exposed versus non-exposed).

Characteristic	Some exposure^a^ (*N* = 510)	No exposure^a^ (*N* = 170)	*P*-value^b^
Child age (yrs.)—mean (std)	12.50 (2.52)	11.98 (2.30)	0.0191
Caregiver age (yrs.)—mean (std)	40.49 (9.45)	38.16 (8.63)	0.0048
Child race/ethnicity—no. (%)			0.9619
African American	374 (73.62)	124 (73.81)	
Other	134 (26.38)	44 (26.19)	
Child biological sex—no. (%)			0.3521
Female	270 (52.94)	83 (48.82)	
Male	240 (47.06)	87 (51.18)	
Caregiver education—no. (%)			0.7971
Less than high school (HS) degree	58 (11.46)	19 (11.31)	
HS/some college/Trade	346 (68.38)	119 (70.83)	
Associate degree or more	102 (20.16)	30 (17.86)	
Caregiver BMI—mean (std)	33.37 (8.81)	32.65 (10.16)	0.4341
EBT/Bridge or SNAP—no. (%)			0.2060
Yes	311 (61.22)	112 (66.67)	
No	197 (38.78)	56 (33.33)	
Free/reduced school lunch—no. (%)			0.8090
Yes	297 (58.46)	100 (59.52)	
No	211 (41.54)	68 (40.48)	
City of residence—no. (%)			0.4889
Flint	382 (75.35)	131 (77.98)	
Other	125 (24.65)	37 (22.02)	
Caregiver’s overall perceptions of pediatric health care (score)—mean (std)	85.81 (15.36)	79.84 (18.07)	<0.0001

^a^Some cross-table cell counts do not add up to the marginal counts due to missing data in the demographic characteristics.

^b^*P*-values are generated using standard chi-square and student’s *t*-tests.

### Exposure by Number of Prescriptions Received

As shown in Table 2, children who were exposed to the FVPP recorded significantly lower blood pressure percentile measures when compared to unexposed children (63.273 versus 75.060 for SBP; 71.472 versus 77.548 for DBP). For the discretized versions, a significantly lower proportion of children in the exposed group recorded elevated blood pressure measurements (blood pressure above 90%) when compared to their counterparts in the unexposed group (0.141 versus 0.343 for SBP; 0.199 versus 0.344 for DBP; and 0.286 versus 0.531 overall). Differences in BMI percentiles and elevated BMI proportions (>95%) among youth by exposure group were not significant.

**Table 2:** Potential outcome means of clinical outcomes for both exposure groups, risk, and mean differences, with 95% confidence intervals.

Clinical outcomes	Causal effect estimates and confidence intervals^a^		
	Exposed (one or more FVRx)	Unexposed (no FVRx)	Average treatment effect (risk or mean difference)
Child weight status, *n* = 652			
BMI percentile	75.323 (72.706, 77.716)	77.159 (72.888, 81.473)	−1.836 (−6.747, 3.413)
Obesity (BMI percentile >95%)	0.393 (0.347, 0.439)	0.419 (0.339, 0.500)	−0.026 (0.117, 0.071)
Child SBP status, *n* = 651			
SBP percentile	63.273 (61.027, 65.433)	75.060 (71.565, 78.613)	−11.787 (−15.84, −7.619)^c^
Elevated SBP (SBP% > 90%)	0.141 (0.110, 0.173)	0.343 (0.270, 0.423)	−0.202 (−0.287, −0.122)^c^
Child DBP status, *n* = 651			
DBP percentile	71.472 (69.592, 73.353)	77.548 (73.983, 80.948)	−6.076 (−10.117, −2.133)^c^
Elevated DBP (DBP% > 90%)	0.199 (0.164, 0.236)	0.344 (0.264, 0.446)	−0.145 (−0.255, −0.058)^c^
Elevated overall BP^b^, *n* = 653	0.286 (0.243, 0.328)	0.531 (0.447, 0.616)	−0.245 (−0.338, −0.152)^c^

^a^The estimates of the POM and ATE are generated by balancing the two exposures across the distribution of the following potential confounders: child’s age, child’s gender, child’s race (African American versus others), caregiver’s education, caregiver’s age (age < 38 years vs. age ≥ 38 years), caregiver’s BMI (BMI < 30 vs. BMI ≥ 30), participation of any member of the household to any supplemental nutrition assistance program or free/reduced school lunch (yes vs. no), city of residence (Flint vs. other) and caregiver’s overall perceptions of child health care (score <90 vs. score ≥ 90).

^b^Normal BP: <90th percentile for children under 13 and Normal BP: <120/<80 mm Hg for children 13 and above.

^c^Significant at 5% level.

As shown in Table 3, youth who were exposed to the FVPP reported significantly greater levels of physical activity when compared to unexposed youth (mean difference in point value of 2.216). Albeit not reaching statistical significance, a higher proportion of caregivers whose children were exposed to the FVPP reported high/moderate household food security when compared to caregivers whose children were not exposed to the FVPP (0.679 versus 0.631). No appreciable differences were observed for mean daily consumption of fruits and vegetables as self-reported by youths; however, mean estimates favored children in the exposed group.

**Table 3:** Potential outcome means for both exposure groups, risk, and mean differences, with 95% confidence intervals.

Food security, physical activity, and FV intakes	Causal effect estimates and confidence intervals^a^		
	Exposed (one or more FVRx)	Unexposed (no FVRx)	Average treatment effect (risk or mean difference)
Food security perception (FSP), *n* = 659			
Household high/moderate FSP	0.679 (0.639, 0.724)	0.631 (0.557, 0.711)	0.048 (−0.039, 0.134)
Child physical activity score, *n* = 652	21.783 (21.068, 22.591)	19.567 (18.211, 20.993)	2.216 (0.653, 3.696)^c^
Mean daily intake of fruits and vegetables among youth, *n* = 659			
Total fruits (CE^b^)	1.437 (1.324, 1.555)	1.275 (1.119, 1.441)	0.162 (−0.034, 0.346)
Total whole fruits (CE^b^)	0.694 (0.632, 0.756)	0.619 (0.516, 0.728)	0.074 (−0.066, 0.190)
Total vegetables (CE^b^)	1.063 (0.981, 1.146)	1.016 (0.882, 1.167)	0.047 (−0.134, 0.199)
Vegetable excl. potatoes/legumes (CE^b^)	0.694 (0.629, 0.750)	0.654 (0.564, 0.767)	0.040 (−0.094, 0.143)
Total legumes (CE^b^)	0.057 (0.048, 0.069)	0.048 (0.036, 0.063)	0.009 (−0.008, 0.026)
Other fruits (CE^b^)	0.128 (0.111, 0.149)	0.114 (0.089, 0.142)	0.014 (−0.019, 0.045)
Total fruit juice (CE^b^)	0.617 (0.556, 0.681)	0.539 (0.446, 0.635)	0.078 (−0.031, 0.189)

^a^The estimates of the POM and ATE are generated by balancing the two exposures across the distribution of the following potential confounders: child’s age, child’s gender, child’s race (African American versus others), caregiver’s education, caregiver’s age (age < 38 years vs. age ≥ 38 years), caregiver’s BMI (BMI < 30 vs. BMI ≥ 30), participation of any member of the household to any supplemental nutrition assistance program or free/reduced school lunch (yes vs. no), city of residence (Flint vs. other) and caregiver’s overall perceptions of child health care (score <90 vs. score ≥ 90).

^b^*CE* cup equivalents.

^c^Significant at 5% level.

### Exposure by FVPP duration

As shown in Table 4, children with high exposure to the FVPP recorded blood pressure percentile measures that were significantly lower than those in the low exposure group (60.053 versus 74.868 for SBP; 70.242 versus 76.862 for DBP). For the discretized versions, a significantly lower proportion of children in the high exposure group recorded elevated blood pressure measurements (blood pressure above 90%) when compared to their counterparts in the low exposure group (0.090 versus 0.333 for SBP; 0.178 versus 0.310 for DBP; and 0.240 versus 0.500 overall). Differences in BMI percentiles among youth by exposure group were not significant.

**Table 4:** Potential outcome means of clinical outcomes for both exposure groups, risk, and mean differences, with 95% confidence intervals.

Clinical Outcomes	Causal effect estimates and confidence intervals^a^		
	High exposure (exposure ≥24 months)	Low exposure (exposure <24 months)	Average treatment effect (risk or mean difference)
Child weight status, *n* = 652			
BMI percentile	75.345 (72.280, 78.097)	76.053 (72.858, 79.456)	−0.709 (−5.13, 3.716)
Obesity (BMI percentile >95%)	0.385 (0.333 0.431)	0.414 (0.351, 0.476)	−0.029 (−0.104, 0.049)
Child SBP status, *n* = 651			
SBP percentile	60.053 (57.373, 62.589)	74.868 (72.338, 77.561)	−14.815 (−18.794, −11.110)^c^
Elevated SBP (SBP% > 90%)	0.090 (0.059, 0.120)	0.333 (0.278, 0.390)	−0.242 (−0.303, −0.178)^c^
Child DBP status, *n* = 651			
DBP percentile	70.242 (67.927, 72.268)	76.862 (74.656, 78.918)	−6.620 (−9.865, −3.509)^c^
Elevated DBP (DBP% > 90%)	0.178 (0.138, 0.223)	0.310 (0.258, 0.368)	−0.132 (−0.197, −0.066)^c^
Elevated overall BP^b^, *n* = 653	0.240 (0.197, 0.286)	0.500 (0.437, 0.559)	−0.260 (−0.334, −0.186)^c^

^a^The estimates of the POM and ATE are generated by balancing the two exposures across the distribution of the following potential confounders: child’s age, child’s gender, child’s race (African American versus others), caregiver’s education, caregiver’s age (age < 38 years vs. age ≥ 38 years), caregiver’s BMI (BMI < 30 vs. BMI ≥ 30), participation of any member of the household to any supplemental nutrition assistance program or free/reduced school lunch (yes vs. no), city of residence (Flint vs. other) and caregiver’s overall perceptions of child health care (score <90 vs. score ≥ 90).

^b^Normal BP: <90th percentile for children under 13 and Normal BP: <120/<80 mm Hg for children 13 and above.

^c^Significant at 5% level.

As shown in Table 5, youth who were exposed to the FVPP for at least 24 months (high exposure) reported significantly greater levels of physical activity when compared to youth in the low exposure group (mean difference in point value of 3.206). Albeit not reaching statistical significance, a higher proportion of caregivers whose children were in the high exposure group reported high/moderate household food security when compared to caregivers whose children were less exposed to the FVPP (0.693 versus 0.636). No appreciable differences were observed for mean daily consumption of fruits and vegetables as self-reported by youths.

**Table 5:** Potential outcome means for both exposure groups, risk, and mean differences, with 95% confidence intervals.

Food security, physical activity, and FV intakes	Causal effect estimates and confidence intervals^a^		
	High exposure (exposure ≥24 months)	Low exposure (exposure <24 months)	Average treatment effect (risk or mean difference)
Food security perception (FSP), *n* = 659			
Household high/moderate FSP	0.693 (0.643, 0.741)	0.636 (0.584, 0.687)	0.057 (−0.011, 0.131)
Child physical activity score, *n* = 652	22.817 (21.926, 23.770)	19.611 (18.665, 20.670)	3.206 (1.719, 4.397)^c^
Mean daily intake of fruits and vegetables among youth, *n* = 659			
Total fruits (CE^b^)	1.407 (1.281, 1.533)	1.376 (1.234, 1.534)	0.031 (−0.159, 0.228)
Total whole fruits (CE^b^)	0.691 (0.620, 0.769)	0.658 (0.584, 0.753)	0.033 (−0.080, 0.142)
Total vegetables (CE^b^)	1.013 (0.929, 1.128)	1.081 (0.983, 1.207)	−0.068 (−0.229, 0.072)
Vegetable excl. potatoes/legumes (CE^b^)	0.663 (0.601, 0.747)	0.711 (0.645, 0.792)	−0.048 (−0.156, 0.051)
Total legumes (CE^b^)	0.055 (0.044, 0.068)	0.056 (0.045, 0.069)	−0.001 (−0.017, 0.016)
Other fruits (CE^b^)	0.130 (0.109, 0.156)	0.117 (0.097, 0.138)	0.013 (−0.016, 0.044)
Total fruit juice (CE^b^)	0.592 (0.527, 0.665)	0.598 (0.523, 0.694)	−0.006 (−0.109, 0.100)

^a^The estimates of the POM and ATE are generated by balancing the two exposures across the distribution of the following potential confounders: child’s age, child’s gender, child’s race (African American versus others), caregiver’s education, caregiver’s age (age < 38 years vs. age ≥ 38 years), caregiver’s BMI (BMI < 30 vs. BMI ≥ 30), participation of any member of the household to any supplemental nutrition assistance program or free/reduced school lunch (yes vs. no), city of residence (Flint vs. other) and caregiver’s overall perceptions of child health care (score <90 vs. score ≥ 90).

^b^*CE* cup equivalents.

^c^Significant at 5% level.

## Discussion

In this observational study, researchers used causal inference analyses to compare measures of dietary intake, food security, physical activity, weight status, and blood pressure between pediatric patients with varying levels of exposure to an FVPP. Results indicated notable differences in blood pressure and physical activity between exposure groups. Blood pressure was lower and physical activity was higher among young patients with greater exposure to the FVPP.

Young participants in the current study predominantly identified as African American and resided in Flint, Michigan, a low-income urban community with persistently high rates of child poverty.^49^ Central to study findings were significantly lower blood pressure readings among children with greater exposure to the FVPP. Pediatric hypertension is associated with subclinical atherosclerosis and target organ damage in childhood and can increase the risk of cardiovascular disease in adulthood.^50^ Uncontrolled hypertension is prevalent among African Americans, who disproportionately suffer from its consequences to a greater extent than individuals in other racial groups.^51^ Moreover, African Americans tend to not only develop high blood pressure at younger ages,^52^ but are then less likely than other racial groups to adhere to antihypertensive medication treatment plans.^53^ Current evidence suggests that when compared to unexposed children, those in the exposed group (who were significantly older) would have presented with higher blood pressure values.^52^ However, results from the current study suggest that children with exposure to the pediatric FVPP had appreciably lower systolic and diastolic blood pressure. If replicated, these findings may indicate an important health-promoting effect of FVPP participation, with respect to blood pressure values among young patients. Moreover, the current program, which was designed in partnership with pediatricians to allow ease of implementation within busy clinical settings, provides a tangible resource to actively support chronic disease prevention strategies during critical periods of development.

Levels of physical activity were also consistently higher among children with greater exposure to the FVPP. The “transfer effect” may have occurred in the current project such that the FVPP, which was designed to promote one health behavior (fruit and vegetable intake), facilitated changes in another health behavior (physical activity). This phenomenon has been noted in previous research related to nutrition and physical activity interventions.^54,55^ Although education was not standardized in the current pediatric FVPP, fruit and vegetable prescriptions were consistently provided to families with guidance from pediatricians regarding the importance of dietary choices and engagement in healthy behaviors. It is, therefore, plausible that this FVPP may have impacts on physical activity that may, in turn, partially explain the lower blood pressure among exposed youth.^56,57^ It also cannot be ruled out that there may have been unmeasured confounding factors between the exposure groups that contributed to differences in physical activity.

Findings related to dietary intake among youth in relation to exposure to the current FVPP contradict a growing body of literature that indicates a positive effect of such programs on diets of children, particularly in relation to fruit and vegetable intake.^18,19,21–23^ It is worth noting that diet and food security measures were likely impacted by local, state, and federal programs that provided significant resources to families during the COVID-19 pandemic and while data collection for the current study occurred. Previous research has indicated most people living in the US experienced improved food security and food choice healthfulness resulting from vigorous federal, state, city, and community responses to the pandemic.^58,59^ For example, the Pandemic-Electronic Benefit Transfer was available from September 2021 through June 2022 for children who were eligible for free or reduced school lunch but not receiving due to school closures during COVID-19. Grab-and-go meals were also widely available for students throughout Flint, and surrounding Genesee County, during the period of data collection. And, until March 2023, the Supplemental Nutrition Assistance Program (SNAP) provided additional benefits to households with allowances that substantially increased food dollars within households. Research has confirmed the success of these programs in reaching caregivers while also supporting food access among youth.^60,61^ Although participation in these additional programs was not tracked in the current study, the majority of study participants reported participation in SNAP and free/reduced-priced lunch. It is, therefore, reasonable to suspect that these additional allowances, designed to assist families during the COVID-19 pandemic, had a direct impact on youth diets and household food security that was not considered.

Furthermore, accurately assessing dietary intake is complex, especially among youth. This process proves challenging for many reasons, including difficulty recalling foods consumed, errors in estimating portion sizes, overall misreporting, and day-to-day variation in intake.^62^ Previous research has suggested that intake of fruits and vegetables among youth in Flint, Michigan, is markedly low.^11,63^ With the knowledge that a minimum of three days of dietary recalls for each participant would have been required to accurately estimate total intake of infrequently consumed foods, such as fruits and vegetables,^40,64^ researchers chose to employ a validated food screener. The BKFS was selected specifically because of its low administration burden and ability to assess dietary intake over seven total days.^40^ Still, the only validation study for the BKFS suggested that three days of recall (utilized in the validation study) may be too few to accurately measure intake of foods, such as fruits and vegetables, that are infrequently consumed.^65^ It is, therefore, possible that the dietary assessment tool used in the current study was insufficient to detect differences in dietary intake of participating children. Future longitudinal research with the low exposure group will assess, through 24-hour dietary recalls together with BKFS data, whether changes in consumption of fruits and vegetables occur over time.

Limitations of the current study should be acknowledged. First, the sample was specific to one geographic location and restricted to only those who spoke English. Although study participants were demographically similar at baseline, it cannot be ruled out that there may have been unmeasured confounding factors between the exposure groups that contributed to differences in physical activity and blood pressure. Next, there was no measure of baseline blood pressure among young participants who had previous exposure to the FVPP. Although prescription distribution was carefully tracked in the current study, prescription redemption as a measure of engagement was not assessed. Finally, and as previously mentioned, there was no measure of engagement in COVID-related relief programs that may have impacted dietary patterns and household food security.

## Conclusions

The current study is among the first to fill gaps in evidence about the potential effects of FVPPs directed at youth. Unique in its focus on the critical role of fruits and vegetables in disease prevention, this study extends evaluation of the current pediatric FVPP beyond feasibility and suggests the potential of the program to influence physical activity and blood pressure of participating youth. If replicated, findings may indicate novel health-promoting effects of pediatric FVPPs.

## Supplementary information

CONSORT Checklist

## Data Availability

The datasets generated and analyzed during the current study will be available in the National Institutes of Health-supported data repository.
